# Modelling lifetime cost consequences of ReSTOR^® ^in cataract surgery in four European countries

**DOI:** 10.1186/1471-2415-8-12

**Published:** 2008-07-15

**Authors:** Antoine Lafuma, Gilles Berdeaux

**Affiliations:** 1 Cemka, 43 boulevard du maréchal Joffre, F-92240 Bourg-la-Reine, France; 2 Alcon France, 4, rue Henri Sainte-Claire Deville, F-92563 Rueil-Malmaison, France

## Abstract

**Background:**

To compare the lifetime costs of liberating patients from spectacles after cataract surgery by implanting the multifocal intraocular lens (IOL) 'ReSTOR^®^' *versus *monofocal IOLs in France, Italy, Germany and Spain.

**Methods:**

A Markov model was created to follow patient cohorts from cataract surgery until death. Prevalence rates of patients not needing spectacles after cataract surgery were obtained from clinical trials. Resource utilisation included implant surgery, IOLs, spectacles, visits to ophthalmologists and eye centres, transport, and time lost by patients. Economic perspectives were those of Society and Sickness Funds (SFs).

**Results:**

The mean number of spectacles purchased after ReSTOR^® ^was 1.34–1.61 and after monofocal IOLs 6.05–7.27. From the societal perspective, total cost estimates discounted by 3% were between €3,551 and €4,052 with ReSTOR^® ^compared to €3,989 and €5,548 with monofocal IOLs. Undiscounted savings related to ReSTOR^®^ ranged from €815 to €2,164. From the SFs' perspective total cost estimates discounted by 3% were between €2,150 and €2,524 with ReSTOR^® ^compared to €2,324 and €2,610 with monofocal IOLs. Savings related to ReSTOR^®^, once costs discounted, ranged from €61 to €219. Discount and spectacle freedom prevalence rates were the most sensitive parameters.

**Conclusion:**

The bulk of the savings related to ReSTOR^®^ were realized outside the SF. From both a societal and SF perspective, savings, after a 3% discounting, achieved by liberating patients from spectacles counterbalanced the initially higher cost of ReSTOR^®^. ReSTOR^® ^is a cost saving alternative to spectacles for patients requiring cataract surgery.

## Background

Typical senile cataract progresses slowly and can cause vision loss if untreated. An estimated 20.5 million (17.2%) Americans older than 40 years have a cataract in at least one eye [1].

While cataract is the leading cause of blindness in the world [2,3] most developed Western populations have access to cataract surgery (*e.g*., 6.1 million American citizens (5.1%) have pseudo-phakia/aphakia). The total number of Americans with cataract is predicted to increase to 30.1 million by 2020 and of these 9.5 million are expected to have pseudo- phakia/aphakia [1].

More than 80% of patients regain good best-corrected visual acuity (≥ 8/10) after cataract surgery, depending on other ocular pathology and the duration of follow-up [4-8].

Traditional intraocular lenses are monofocal and after implantation most patients need spectacles for at least near vision. Multifocal IOLs are meant to free patients from spectacles after presbyopia or cataract surgery by applying the principle of simultaneous vision [9]. Improvements in intraocular lens technology have permitted cataract patients to be implanted with multifocal IOLs providing better visual acuity at various distances and a degree of spectacle independence [10]. Early MIOLs were initially associated with loss of clarity and accommodation, reduced contrast sensitivity, and complaints of halos and glare. Today, MIOLs produce functional near and distance vision in everyday practice and an acceptable level of patient satisfaction [11-25]. Recently, a new apodised IOL (ReSTOR^®^) was marketed that combines efficacy at both near and distant vision (80% of the patients never wear spectacles).

According to Vitale *et al*. [26] more than 110 million Americans either could, or do, achieve normal vision after refractive correction. However, spectacle prescriptions incur costs for both patients and health insurance providers. The annual direct cost of simply correcting impaired distance vision was at least $3.8 billion of which $780 million concerned persons aged > 65 years. To our knowledge no data have been published on the costs associated with wearing spectacles after cataract surgery apart from a survey conducted by Cuq *et al*. [27] in France, Germany, Italy and Spain, which found costs varying from €230 (Spain) to €579 (France). Cost savings have been estimated for 'laser-assisted *in situ *keratomileusis' (LASIK) [28], but to date no lifelong data have been published for multifocal IOLs.

The aim of this economic analysis was to compare the lifetime costs and consequences of liberating patients from spectacles by implanting the multifocal IOL 'ReSTOR^®^' *versus *monofocal IOLs after cataract surgery.

## Methods

This economic study used a Markov model to estimate the lifetime cost consequences for Society and Sick Funds (SFs) in four European countries (France, Germany, Italy and Spain) after implanting monofocal IOLs, multifocal IOLs or ReSTOR^® ^bilaterally during cataract surgery. TreeAge software version 4.0 was used to build a Markov model simulating cohorts of patients implanted with identical IOLs into both eyes during cataract surgery at age 70 years, followed until death or age 100 years.

**Figure 1:**
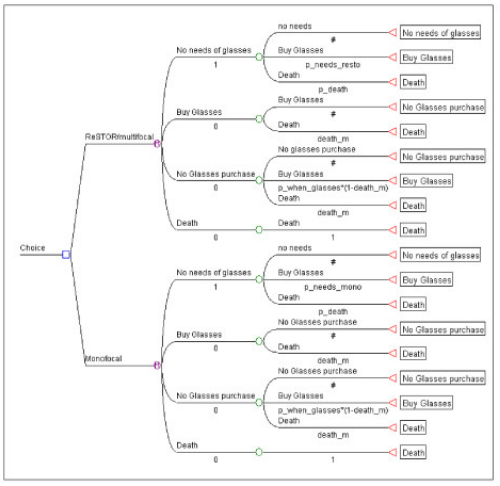
Model schema.

Four Markov states were created (Figure 1) as follows: "No needs of glasses", "Buy glasses", No glasses purchase" and "Death", the absorbing state. Thus, after cataract surgery patients presented with one of two possible health states, either "No needs of glasses" or "Buy glasses". The frequency rate of "No needs of glasses" was derived from controlled clinical trials. Patients starting in the "Buy glasses" state were included and remained in the "No glasses purchase" until they bought spectacles, after which they joined the "Buy glasses" state for one cycle.

The duration of a cycle was 1 month and patients were eliminated at death or on reaching 100 years of age. National mortality statistics were used for life expectancy [29-32].

The following assumptions were made when constructing the model:

• A patient freed from spectacles by an MIOL would remain spectacle free for life (MIOLs are implanted for decades of use)

• The spectacle type prescribed after a monofocal IOL implant was assumed, conservatively, to be similar to that after an MIOL (whereas monofocal spectacles are more frequently provided after MIOLs)

• Outside the near vision benefit, the risk-benefit ratio with MIOL surgery was made similar to that for monofocal IOLs

• Patients given excimer laser surgery following MIOL implantation were excluded, as stipulated in the protocol that established the ReSTOR^® ^spectacle independence rate

• Age was associated with death only, and not with the cost of spectacles.

The base-case analysis compared two groups of patients, patients implanted with ReSTOR^® ^in both eyes *versus *patients with monofocal IOLs in both eyes.

A three-way sensitivity analysis was performed on the primary sensitive parameters, *i.e*. discount rates (0%, 3% and 5%), multifocal IOL premiums (€0, €250 and €500) and prevalence of spectacle independence (20%, 40%, 60% and 80%).

## Efficacy and resource utilisation

In a study submitted to the FDA [33], ReSTOR^® ^achieved 80% rates of spectacle independence for both distance and near vision, figures much higher than reported previously for other multifocal implants. ReSTOR^® ^also provided qualitatively better visual acuity and spectacle independence, with tolerable unwanted photic phenomena [22,23,34] from both the clinical and patients' perspectives. In the base-case analysis, spectacle freedom prevalence rates were fixed at 80% for ReSTOR^® ^and 10% for monofocal implants.

A survey was carried out across four countries to estimate the costs of wearing spectacles after cataract surgery. The patient sample [27] was recruited by ophthalmologists and given a questionnaire to answer. Resources consumed and the associated costs of cataract surgery were itemised, as follows: (1) surgical operation (both eyes); (2) two implants (monofocal or ReSTOR^®^); (3) time spent on surgery (including transport); and (4) other transport to the clinic. Resources consumed by a patient needing spectacles were, as follows: (1) consultation with ophthalmologists or optometrists for prescriptions; (2) transport and time spent during the visit; (3) visits to the optic centres; (4) time spent in choosing and collecting spectacles; and (5) transport to the ophthalmologist's or optometrist's office. An annual rate for spectacle replacement was estimated from the survey.

In addition to the model, the following resources were consumed periodically by patients wearing spectacles: (1) visits to an optic centre for frame maintenance; (2) time spent on the visit; (3) cleaning devices purchased; and (4) transport to the optic centre.

## Costing

Results are expressed as 2006 euros. A discount rate of 3% was fixed for the base-case analysis.

National tariffs and estimates were used to cost medical resources consumed (Table 1). The following unit costs were estimated from national tariffs and available sources:

**Table 1:** Unit costs according to country and type of resource consumed (2006 €)

**Item**	**France**		**Germany**		**Italy**		**Spain**	
	
	**Society**	**NHS**	**Society**	**NHS**	**Society**	**NHS**	**Society**	**NHS**
Cataract surge'ry								
• Surgery [35-38]	1,145.9	1,145.9	1,250	1,250	1,106	1,106	1,050	1,050
• ReSTOR^® ^premium^1^	500	0	500	0	500	0	500	0
• Monofocal IOLs	In DRG	In DRG	In DRG	In DRG	In DRG	In DRG	In DRG	In DRG
• Other multifocal IOLs^2^	[0–500]	0	[0–500]	0	[0–500]	0	[0–500]	0

Spectacles [25,34]	578.9	19.71	387.6	0	310.5	0	230.2	0
Unit cost of cleaning	3		3		3		3	
Visit [39-42]								
• ophthalmologist	25	16.50	57.78	23.75	100	12.91	60	60
• optometrist	-	-	0	0	0	0	40	0
Mean cost *per *km [43-46]								
• visit/implant	0.39	-	0.27	-	0.31	-	0.46	-
• optic centre	0.31	-	0.23	-	0.31	-	0.45	-
Cost of work/hour [47]	24.7	-	26.22	-	21.39	-	14.75	-

^1 ^Fixed arbitrarily at €500

^2 ^Analysed by sensitivity analysis

(a) according to a European survey [27] most people spend from €200 to €400 for a pair of spectacles, except in France where the majority pays more than €500. Spectacle costs were not reimbursed, except in France [35]. In France, national health service (NHS) tariffs vary according to age (above or below 18 years) and the degree of optical correction. For subjects above 18 years lenses are reimbursed at 65% and frames are reimbursed at 65% of a fixed tariff of €2.84;

(b) the average cost of cataract surgery varied from €1,050 in Spain to €1,250 in Germany, including the cost of a monofocal intraocular implant [36-39];

(c) for the societal perspective, an estimated €500 was added to the cost of cataract surgery with ReSTOR^®^, as a premium for spectacle independence; in the absence of published data, this figure was estimated from our knowledge of the market.

(d) ophthalmologists' consultation fees varied from €25 in France to €100 in Italy, with optometrists' costs influenced by official regulations, *i.e*. in Italy and Germany optometry is not a recognised profession and many optometrists do not charge for a visit, whereas in Spain optometrists' costs are regulated at €40 without NHS reimbursements [37,40-43];

(e) spectacle cleaning materials vary widely in cost, according to package size, brand and type of accessories (spray, cloths and chains), hence an approximated average cost of €3 was applied to the present analysis;

(f) as costs per kilometre and type of transportation differed between countries (taxis from €0.9 in France to €1.80 in Spain, with subway and bus costs similar at about €0.20-€0.30, depending on the ticket and distance covered) different internet sources [44-47] were solicited to obtain an approximated average cost of €0.40 per kilometre weighted by the proportion of subjects using the various types of transportation [27]; distances to ophthalmologists varied from 11.9 to 29.1 km, and distances to optic centres from 6.5 to 13.4 km [27].

(g) time spent was economically valued using estimated hourly earnings published by the European Union Statistical Office [48].

## Results

According to the Markov model and using national mortality statistics for each country, the average life expectancy of patients aged 70 years in the four European countries ranged from 13.8 to 15.0 years, reflecting differences in general life expectancy from 78.4 years in Germany to 80.5 years in Spain [49]. The average estimated duration of spectacle wear with monofocal IOLs was 12.4 to 13.5 years, which was more than 4× the duration following ReSTOR^® ^implants (2.8 to 3.0 years depending on the country).

Table 2 shows average resource consumption per patient according to the type of IOL implanted and the country. Consistent with the average duration of spectacle dependence patients implanted with monofocal lenses consumed 4× more on spectacles, visits to ophthalmologists, transportation and cleaning devices, and devoted about three additional full weeks of life to dealing with their visual acuity compared to ReSTOR^® ^patients.

**Table 2:** Average resources consumed in the Markov model period according to ReSTOR^®^, monofocal implants and countries

**Item**	**France**		**Germany**		**Italy**		**Spain**	
	
	**ReSTOR**^®^	**Monofocal**	**ReSTOR**^®^	**Monofocal**	**ReSTOR**^®^	**Monofocal**	**ReSTOR**^®^	**Monofocal**
Number of spectacles	1.34	6.05	1.44	6.49	1.61	7.27	1.45	6.52
Number of units purchased to clean spectacles	7.5	33.9	4.55	20.46	7.02	31.59	2.97	13.34
Visit to ophthalmologist to correct visual acuity	1.34	6.05	1.28	5.77	1.34	6.03	0.94	4.24
Visit to optometrist to correct visual acuity	-	-	-	-	0.27	1.24	0.5	2.3
Transportation ophthalmologist (km)	195	468	82	202	182	466	150	370
Transportation optic centre (km)	127	573	87	390	180	808	157	705
Time spent to care for visual acuity (h)	44.5	57.0	43.9	53.9	45.2	60.1	43.6	52.9

Base-case scenario: cost of ReSTOR^® ^= €500; discount rate = 3%; patients freed from spectacles = 80% after ReSTOR^® ^*versus *10% after monofocal IOLs.

Table 3 compares the estimated societal costs of a patient implanted with monofocal IOLs or ReSTOR^® ^according to our base-case scenario. In all countries, the incremental cost of ReSTOR^® ^was less than the savings achieved with spectacle independence. Major savings related directly to the cost of spectacles, followed by time spent and transportation. Lastly, ReSTOR^® ^enabled cost savings even in the country where spectacles cost the least (Spain, €230.2). Lifelong 3% discounted savings ranged from €547 to €1,741.

**Table 3:** Cost consequences (€) according to ReSTOR^® ^and monofocal IOLs per country from the societal perspective

**Item**	**France**		**Germany**		**Italy**		**Spain**	
	
	**ReSTOR**^®^	**Monofocal**	**ReSTOR**^®^	**Monofocal**	**ReSTOR**^®^	**Monofocal**	**ReSTOR**^®^	**Monofocal**
Surgery including the IOL	3,292	2,292	3,500	2,500	3,212	2,212	3,100	2,100
Spectacles	615	2,766	447	2,009	394	1,772	334	1,501
Clean spectacles	17.5	78.5	11	48	16	73	9	40
Visit to correct visual acuity	27	119	59	267	105	474	77	346
Transportation	100	292	36	117	94	314	140	487
**Total without time spent**	**4,051**	**5,548**	**4,052**	**4,941**	**3,821**	**4,844**	**3,551**	**3,989**

Time spent	1,081	1,324	1,134	1,344	947	1,197	635	744
**Total including time spent**	**5,132**	**6,872**	**5,186**	**6,285**	**4,769**	**6,041**	**4,186**	**4,733**

**Difference **	**-1,741**	**Ref**	**-1,099**	**Ref**	**-1,272**	**Ref**	**-547**	**Ref**

Base-case scenario: cost of ReSTOR^® ^= €500; discount rate = 3%; patients freed from spectacles = 80% after ReSTOR^® ^*versus *10% after monofocal IOLs, Ref: Reference

Table 4 presents the estimated NHS costs of a patient with a monofocal IOL, as compared to a ReSTOR^® ^implant, according to our base-case scenario. In contrast to Table 3, it would appear that the NHS is not a major stakeholder as its costs represented less than one-half the costs of society. Nonetheless, savings were realised by all countries which did not reimburse visits for visual acuity control. These savings ranged from 3.1 to 26.5× times less than those realised by society.

**Table 4:** Cost consequences (€) according to ReSTOR^® ^and monofocal IOLs per country according to NHS perspectives

	**France**		**Germany**		**Italy**		**Spain**	
	
**Item**	**ReSTOR**^®^	**Monofocal**	**ReSTOR**^®^	**Monofocal**	**ReSTOR**^®^	**Monofocal**	**ReSTOR**^®^	**Monofocal**
Surgery including the IOL	**2,292**	**2,292**	**2,500**	**2,500**	**2,212**	**2,212**	**2,100**	**2,100**
Spectacles	21	94	0	0	0	0	0	0
Visit to correct visual acuity	17	79	24	110	14	61	50	224

**Total**	**2,330**	**2,465**	**2,524**	**2,610**	**2,226**	**2,273**	**2,150**	**2,324**
**Difference **	**-135**	**Ref**	**-85**	**Ref**	**-48**	**Ref**	**-174**	**Ref**

Base-case scenario: cost of ReSTOR^® ^= €500; discount rate = 3%; patients freed from spectacles = 80% after RESTOR^® ^*versus *10% after monofocal IOLs. Ref: Reference

Tables 5 and 6 describe three-way sensitivity analyses for each economic perspective. After multifocal IOLs it was necessary for the prevalence rate of freedom from spectacles in France to reach about 20% before they became less expensive than monofocal IOLs, There was a 40% rate in Germany and Italy. In Spain, the country with the lowest cost of spectacles, a freedom from spectacle rate of almost 80% was needed in the most extreme situation (i.e. discount rate 5% and €500 premium). From a NHS perspective savings ranged between €6 and €170.

**Table 5:** Cost differences between monofocal IOLs and other multifocal IOLs (€), excluding time spent, according to freedom from spectacle rates, discount rates and IOL prices (societal perspective)

**Spectacle dependence rates**		**Discount Rate = 0%**			**Discount Rate = 3%**			**Discount Rate = 5%**		
		
		**€0**	**€250**	**€500**	**€0**	**€250**	**€500**	**€0**	**€250**	**€500**
**France**										

**% of spectacles after MIOL**	**20%**	-3,164	-2,664	-2,164	-2,497	-1,997	-1,497	-2,182	-1,682	-1,182
	**40%**	-2,260	-1,760	-1,260	-1,784	-1,284	-784	-1559	-1,059	-559
	**60%**	-1,356	-856	-356	-1,070	-570	-70	-935	-435	**65**
	**80%**	-452	**48**	**548**	-357	**143**	**643**	-312	**188**	**688**

**Germany**										

**% of spectacles after MIOL**	**20%**	-2,365	-1,865	-1,365	-1,888	-1,388	-888	-1,658	-1,158	-658
	**40%**	-1,689	-1,189	-689	-1,349	-849	-349	-1,184	-684	-184
	**60%**	-1,014	-514	-14	-809	-309	**191**	-710	-210	**290**
	**80%**	-338	**162**	**662**	-270	**230**	**730**	-237	**263**	**763**

**Italy**										

**% of spectacles after MIOL**	**20%**	-2,580	-2,080	-1,580	-2,023	-1,523	-1,023	-1,759	-1,259	-759
	**40%**	-1,843	-1,343	-843	-1,445	-945	-445	-1,256	-756	-256
	**60%**	-1,106	-606	-106	-867	-367	**133**	-754	-254	**246**
	**80%**	-369	**131**	**631**	-289	**211**	**711**	-251	**249**	**749**

**Spain**										

**% of spectacles after MIOL**	**20%**	-1,815	-1,315	-815	-1,438	-938	-438	-1,258	-758	-258
	**40%**	-1,296	-796	-296	-1,027	-527	-27	-898	-398	**102**
	**60%**	-778	-278	**222**	-616	-116	**384**	-539	-39	**461**
	**80%**	-259	**241**	**741**	-205	**295**	**795**	-180	**320**	**820**

Bold: monofocal IOLs less expensive than multifocal IOLs. Spectacle freedom after monofocal IOLs = 10%.

**Table 6:** Cost differences between monofocal and other multifocal IOLs (€) (multifocal – monofocal) according to different assumptions (Sickness Funds' perspectives)

**Spectacle dependence rates after MIOLs**	**Discount Rate**		
	
	**0%**	**3%**	**5%**
**France**			

**20%**	-170	-135	-118
**40%**	-122	-96	-84
**60%**	-73	-58	-50
**80%**	-24	-19	-17

**Germany**			

**20%**	-209	-166	-145
**40%**	-149	-119	-104
**60%**	-89	-71	-62
**80%**	-30	-24	-21

**Italy**			

**20%**	-61	-48	-41
**40%**	-43	-34	-30
**60%**	-26	-20	-18
**80%**	-9	-7	-6

**Spain**			

**20%**	-219	-174	-152
**40%**	-156	-124	-109
**60%**	-94	-75	-65
**80%**	-31	-25	-22

Spectacle freedom after monofocal IOLs = 10%.

## Discussion

This economic analysis estimated cost consequences until death or age 100 years, of cataract surgery performed at age 70, using ReSTOR^® ^or monofocal implants, in four European countries (France, Germany, Italy and Spain). The economic perspectives were those of society and Sick Funds. Whereas ReSTOR^® ^is a new technology approved for cataract patients, monofocal IOLs today remain widespread as the standard of care during cataract surgery.

Except in France, where reimbursement rates are low, spectacles were not covered by national health services although these countries financed almost 100% of cataract surgery and visits to ophthalmologists for vision correction. Irrespective of the type of implant used, cataract surgery reimbursement always included the price of a standard implant (*i.e*. monofocal IOLs).

Our study was based on national data (mortality and prevalence of cataract), clinical trials, and a survey in all four countries. The concomitant use of clinical trials and national survey data offered good guarantees of internal and external validity, as recommended by most health economics guidelines [50].

With a time horizon of 30 years and a 3% discount rate, ReSTOR^® ^implantation with 20% of patients wearing spectacles after surgery was always more beneficial over monofocal IOLs, regardless of the country. At a incremental cost of €500, ReSTOR^® ^enabled savings from €547 (Spain) to €1,741 (France), relative to monofocal IOLs. From a Sickness Fund perspective, ReSTOR^® ^was always the better strategy since it avoided a significant number of visits to ophthalmologists and optometrists for refraction. The fact that the discount rate significantly modified our results is not surprising due to the long follow-up period built into our model (up to 30 years). Two variables drove the economic benefit of ReSTOR^®^, *i.e*. time and spectacle independence.

We tried to maintain a very conservative approach. The mean number of broken spectacles was calculated for each country from a spectacle survey [27] and we chose the lowest mean rate.

We valorised time spent using hourly earnings even though most of our population was retired. This was appropriate, as patients with spectacles are obliged to spend time caring for them and such time is lost to other use, whether productive, for leisure or something else. Patients may be willing to pay for spectacle care and this should be taken into account. However, valorising wasted time is always a normative exercise which is why we give two total counts, i.e. with and without spectacle cleaning time.

The 80% incidence rate of freedom from spectacles in this model was based on a clinical trial where excimer laser surgery (ELS) was not permitted and so not taken into account. However, ELS is often performed in everyday practice with a consequently higher incidence of freedom from spectacles and greater savings. According to the results in Table 5, a 10% reduction of the spectacle incidence rate in France (discounting = 0%) results in a saving of €452 (time spent excluded). A field study of the costs and consequences of ELS following multifocal lens implantation is needed for a closer approximation to everyday practice.

Non-financial benefits of being free of spectacles after cataract surgery were not evaluated in this analysis. However, in addition to savings with ReSTOR^®^, patients appeared willing to pay for the benefit of spectacle independence. Some ReSTOR^® ^patients reported [51] broader vision (not restricted by a spectacle frame), feelings of well-being, freedom and youthfulness, and improved socialisation, *etc*.

Most of the published literature on ReSTOR^® ^reports clinical outcomes at 6 months, an usually short follow-up duration for cataract surgery clinical trials. This short period is justified by the fact that, apart from posterior capsular opacification [52], most adverse events following cataract surgery arise in the first few months. ReSTOR^® ^is made from an hydrophobic substance that the Cochrane library has found to be associated with a lower PCO incidence rate than hydrophilic materials [53]. Consequently, the mentioned observations support the finding of Lundqvist who showed that subjective and objective visual function 5 years after cataract surgery remained stable in most patients [54]. Lastly, the reader could use the 'spectacle independence rate' variable of our 3-way sensitivity analysis to estimate what would be the long-term impact of resuming spectacle use, should this eventuality occur.

Our analysis has the following limitations: (1) a model cannot replace longitudinal data collection, but the feasibility and economics of such a survey can be questioned; (2) we hypothesised that the prevalence rate of spectacle independence remains constant until the end of a patient's life whereas the known efficacy of ReSTOR^® ^implants, to date, does not yet extend that many years; medical devices vigilance and long term patient follow-up will help confirming this hypothesis or not (3) the external validity of our survey on spectacle costs could be challenged, but the cost structure was coherent across countries [27]; (4) we valued the cost savings of avoided refraction visits after ReSTOR^® ^fully, which may be disputed as refraction could be a marginal reason for an ophthalmic visit. However, avoided visits were not the main driver of ReSTOR^® ^savings (5) the fact that some patients need an excimer laser procedure and/or IOL exchanges were not included in the model.

The reported results are valid at a population level. From an individual's perspective, the cost of spectacles is likely to be subject to some variation, for example, patients purchasing low cost spectacle are not likely to save money. This phenomenon is well identified by our survey [27], where patients accustomed to paying for high cost spectacles were the most willing to spend extra to be freed from spectacles.

Our model showed that savings with ReSTOR^® ^achieved in our four European countries were mainly realised by societies, i.e. by patients. This was explained by the fact that cost shifting occurred entirely outside national health services which paid for cataract surgery, as usual, but not for the costs of refraction visits; whereas patients avoided the costs related to care and refraction treatment. It is worth noting that the costs met by patients exceeded those of national health services. Hence, our results support the case that patients should be allowed complete freedom to control their own budget for refraction correction during cataract surgery according to their economic circumstances, without any economic control of the national health service, provided the procedure has been judged as efficacious and safe. National health services that would deny them this right may be regarded as economically irrational and unfairly interventionist.

## Conclusion

According to our data and model for cataract, bilateral ReSTOR^® ^(2 × €500) implants were always a cost-saving alternative to monofocal IOL implants when viewed from a societal perspective. Our sensitivity analysis, conducted on the three main drivers of uncertainty, showed that any IOL that can provide a spectacle independence prevalence rate > 80% would always yield our average cost savings.

## Competing interests

Dr Gilles Berdeaux was employed by Alcon France. This analysis was supported by an unrestricted grant from Alcon France SA, Rueil-Malmaison, France.

## Authors' contributions

AL and GB participated in the model elaboration, data analysis, interpretation of results and the writing of the manuscript.

## Pre-publication history

The pre-publication history for this paper can be accessed here:


